# Evaluation of alexithymia in patients affected by rheumatoid arthritis and psoriatic arthritis: A cross-sectional study

**DOI:** 10.1097/MD.0000000000013955

**Published:** 2019-01-25

**Authors:** Maria Sole Chimenti, Giulia Lavinia Fonti, Paola Conigliaro, Juna Hitaj, Paola Triggianese, Miriam Teoli, Marco Galluzzo, Marina Talamonti, Barbara Kroegler, Elisabetta Greco, Roberto Perricone

**Affiliations:** a Rheumatology, Allergology and Clinical Immunology, University of Rome Tor Vergata; b Dermatology, University of Rome Tor Vergata.

**Keywords:** alexithymia, pain, psoriatic arthritis, psychiatric comorbidity, rheumatoid arthritis

## Abstract

Rheumatoid arthritis (RA) and psoriatic arthritis (PsA) are chronic autoimmune diseases leading to joint damage, functional limitation, and disability and are typically associated with several comorbidities. Alexithymia is a personality trait characterized by a disregulation of emotion processing and regulation of emotions that involves a dissociation of emotional and physical responses to life events. A broad association between alexithymia and symptoms as depression, inflammation, and pain has been demonstrated. We aimed at evaluate an association among inflammatory arthritis, as RA and PsA, and alexithymia, and a possible link with clinical characteristics and disease activity.

In this cross-sectional study, we enrolled, from January to December 2017, patients affected by RA or PsA referring to the outpatient's clinic of the Rheumatology Unit of the University of Rome Tor Vergata. The 20-item Toronto Alexithymia Scale (TAS-20) was used to assess alexithymia. Disease activity, function, quality of life, and clinimetric indexes were assessed.

A total of 50 RA patients and 51 PsA patients were enrolled. The TAS-20 score showed 38.6% (39/101) patients had alexithymia, 26.7% (27/101) patients were in the borderline of alexithymia and 34.7% (35/101) patients did not have alexithymia. A statistical significant association was observed between alexithymia and inflammatory indices (ESR: *P = *.029, CRP: *P = *.043) and between alexithymia and clinimetric parameters (ptVAS, pVAS, GH, *P < *.0001 for all comparisons). A significant trend of association has been demonstrated between alexithymia and female gender and concomitant steroid therapy. No correlations among variables such as age, duration of disease, and comorbidities and alexithymia status were observed.

This study suggests that alexithymia assessment should be a part of the comprehensive management of RA and PsA patients.

## 1 Introduction

Rheumatoid arthritis (RA) is a chronic autoimmune inflammatory disease characterized by synovitis that leads to destruction of cartilage and bone, functional limitation, and disability.^[1,2]^ Psoriatic arthritis (PsA) is defined as a chronic inflammatory arthritis typically associated with psoriasis.^[3]^ Both are characterized by the presence of a significantly high prevalence of comorbidities, which can involve different organs and tissues, even the psychiatric sphere.^[4]^ The presence of comorbidities should be taken in consideration by physician in order to guarantee an improvement in quality of life and a major adherence to therapies.^[5]^ The presence of psychological distress, especially depression and anxiety, is a well-known aspect of inflammatory arthritis and has been reported as a common comorbidity. In fact, as underlined in the COMORA study, depression is one of the most frequent ones.^[6]^ Moreover, the activation of the innate and acquired immune response not only leads to inflammation in affected organs, but also mediates behavior abnormalities, including fatigue and depression-like symptoms.^[7]^ Arthritis negatively affects not only the physical and social aspects of patients with RA or PsA, but also their psychological well-being.

Alexithymia is conceptualized as a disorder of emotion regulation mechanisms, which involves a dissociation of emotional and physical responses to life events and bodily sensations.^[1,8,9]^ Sifneos coined the term alexithymia, literally “absence of words for emotion” (*a* = lack, *λεξισ* = word, *τυμοσ* = emotions), to describe people who lack the ability to communicate their feelings or have limited imagination.^[10,11]^ Kokkonen and collaborators examined the prevalence of alexithymia and its associations with sociodemographic factors in a population cohort of the Northern Finland. The population consisted of all 12,058 live-born children in the provinces of Lapland and Oulu in Finland and the prevalence of alexithymia was 9.4% in male and 5.2% in female subjects.^[12]^ Due to the strong correlation between altered emotions and chronic diseases, alexithymia has been assessed in patient affected by inflammatory bowel disease^[13]^ or plaque psoriasis,^[14]^ whereas it has been minimally explored in arthritis.

The aim of this cross-sectional observational study was to assess the prevalence of alexithymia in patients with inflammatory arthritis, as RA and PsA, and potential association with demographic variables, clinimetric values, and concomitant treatments.

## 2 Methods

### 2.1 Patients

We enrolled, from January to December 2017, patients affected by RA or PsA, referred to the outpatients’ clinic of the Rheumatology Unit of the University of Rome Tor Vergata, Rome, Italy. Inclusion criteria were ≥18 years of age and a diagnosis of RA made according to the ACR revised criteria for RA^[15]^ or a diagnosis of PsA made according to the CASPAR criteria.^[16]^ Exclusion criteria were: treatment with more than 7.5 mg per die of prednisone or its equivalents, treatment with Apremilast, patient affected by fibromyalgia, psychiatric disorders, central nervous system (CNS) or peripheral nervous system (PNS) diseases or patients who received or were receiving any systemic treatment with psychotropic drugs or cytotoxic medications. For each patient, demographic, clinical data, and treatments performed were collected. The clinical evaluation of joint disease was assessed using tender and swollen joint count and clinimetric values such as pain-visual analog scale (pVAS), patient-visual analog scale (ptVAS), Global Health index (GH), DAS28 (Disease Activity Score 28) for RA and DAPSA (Disease Activity Index for Psoriatic Arthritis) for PsA. Laboratory values of erythrocytes sedimentation rate (ESR; cut off 0–30 mm/min) and C-reactive protein (CRP; 0–5 mg/L) were evaluated.

### 2.2 Instruments

The Toronto Alexithymia Scale (TAS-20) was used to assess alexithymia. The TAS-20 is a self-report questionnaire which consists of 20 questions inherent emotional items. Each question is rated on a 5-point scale ranging from 1 (strongly disagree) to 5 (strongly agree). Patients can obtain a score that comes from 20 points to 100 points. Internationally accepted cut-off values allow to classify patients in 3 different categories, as follows: from 20 to 50 points—nonalexithymic patients, from 51 to 60 points—borderline alexithymic patients and from 61 to 100 points—alexithymic patients.^[17]^ Questionnaires were fulfilled in a dedicate room were privacy was guaranteed. All subjects were instructed from a psychiatrist and completed the Italian translation of the revised 20-item Toronto Alexithymia Scale (TAS-20),^[18–20]^ developed by the usual method of back-translation and cross-validated using confirmatory factor analysis.

The study was carried out according to the Declaration of Helsinki and conducted in accordance with the International Conference on Harmonisation Good Clinical Practice Guidelines. The study protocol was approved by ethic committee of the University of Rome Tor Vergata. All patients provided written informed consent before participating in study-related activities.

### 2.3 Statistical analysis

Continuous variables were presented as means ± standard deviation and were compared using the parametric unpaired *T* test or the nonparametric Mann–Whitney *U* test when appropriate. Categorical variables were presented with absolute frequencies and percentages and were compared using the Chi-squared test or Fisher’ exact test when appropriate. The significance of any correlation was determined by Pearson correlation test or Spearman's rank correlation coefficient where appropriate. *P* values <.05 were considered significant. All statistical analyses were performed using GraphPad Prism version 7 (GraphPad software).

## 3 Results

Around 101 patients, 29 men and 72 women, with a diagnosis of RA (9 men and 41 women) and PsA (20 men and 31 women) were included in this study. The mean age ± SD of RA patients and PsA patients was 59.5 ± 11.5 years (range 22–79) and 52.6 ± 12.8 years (range 27–79), respectively. The disease duration was 6.19 ± 3.82 years for RA and 6.29 ± 3.96 years for PsA. 62 patients (61%) of all the enrolled patients were affected by comorbidities, such as hypertension (26; 25.7%), obesity (1, 1%) diabetes mellitus (8; 7.9%), and osteoporosis (10; 11%). Demographics and clinical characteristics are summarized in Table 1.

**Table 1:**
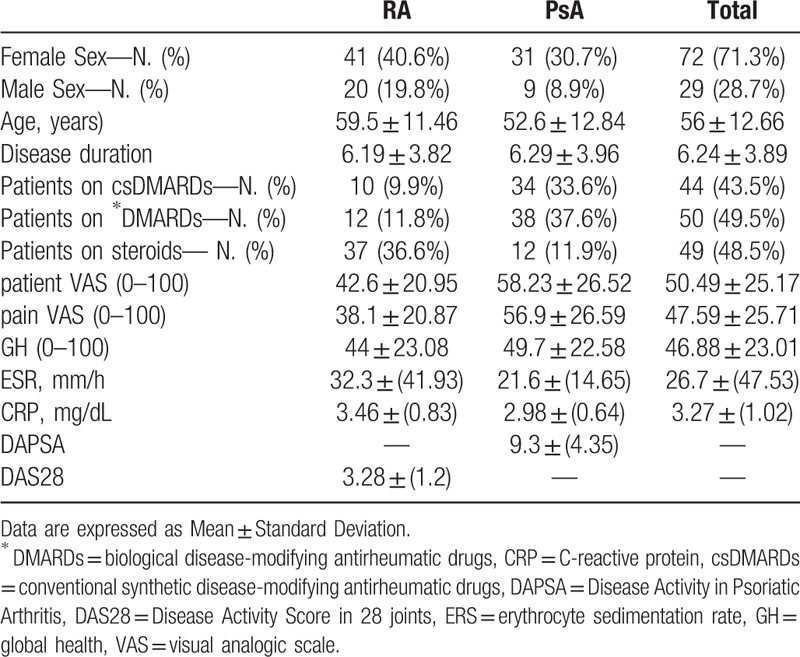
Characteristics, therapies and clinimetric evaluations of the study population.

In the total study population, the TAS-20 score showed that 38.6% (39/101) patients had alexithymia, 26.7% (27/101) patients were in the borderline of alexithymia and 34.7% (35/101) patients did not have alexithymia.

In RA patients’ population, TAS-20 scores showed that 42% (21/50) patients had alexithymia, 24% (12/50) patients were in the borderline area of alexithymia, and 34% (17/50) patients did not have alexithymia.

In PsA patients’ population, TAS-20 score showed that 33.3% (17/51) patients had alexithymia, 29.4% (15/51) patients were in the borderline area of alexithymia and 37.3% (19/51) patients did not have alexithymia.

**Figure 1:**
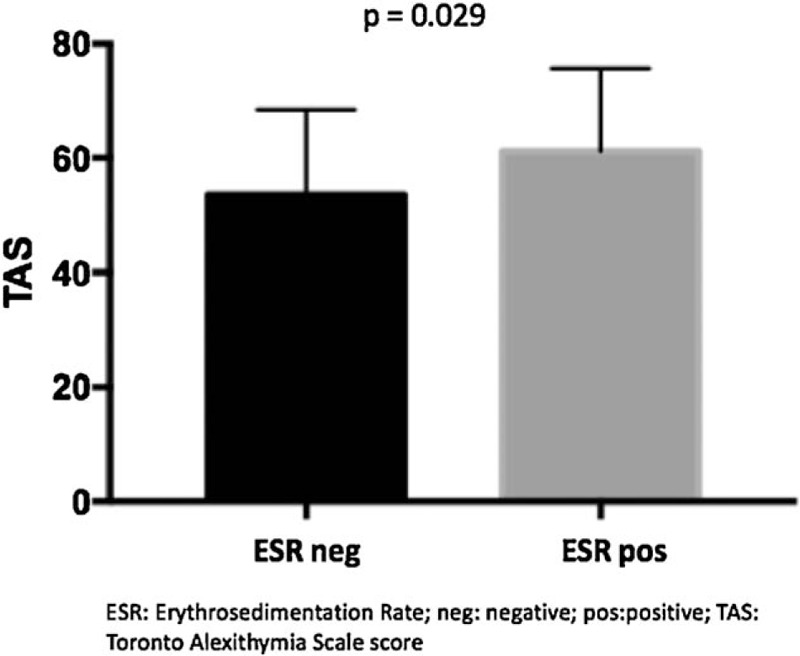
Correlation between TAS scores and ESR values. ESR = erythrocytes sedimentation rate, TAS-20 = 20-item Toronto Alexithymia Scale.

**Figure 2:**
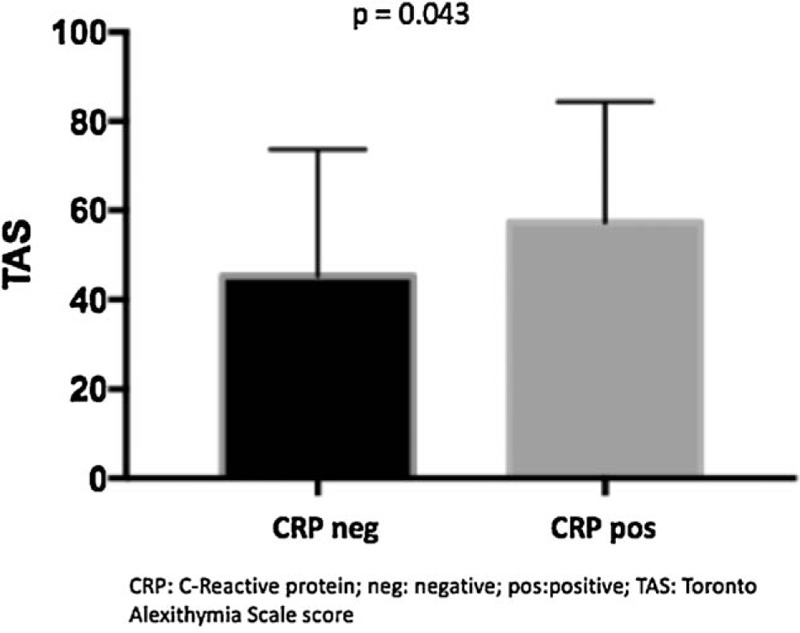
Correlation between TAS scores and CRP values. CRP = C-reactive protein, TAS-20 = 20-item Toronto Alexithymia Scale

**Figure 3:**
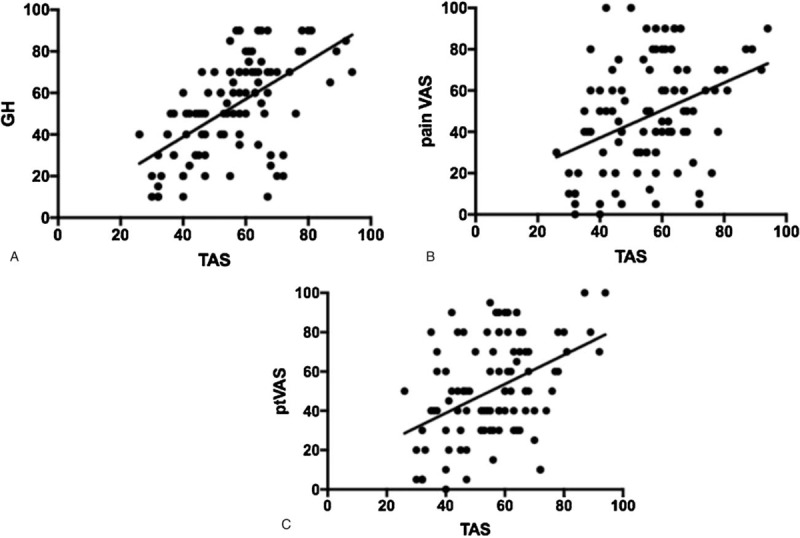
Correlation between TAS and GH (A); correlation between TAS and Pain-VAS (B); correlation between TAS and pt-VAS (C). GH = Global Health index, ptVAS = patient visual analog scale, TAS = Toronto Alexithymia Scale

In addiction, alexithymia TAS-20 values were higher in patients with high values of ESR and CRP. We defined as ESR positive values as levels of ESR > 30 mm/h and CRP positive values were defined as CRP > 0.5 mg/dL. ESR values, of all the study population, directly statistical significantly correlate with the alexithymia values (TAS-20) (*P = *.023) (Fig. 1). Similarly, high CRP, of all the study population, was statistical significantly correlated with TAS-20 scores (*P = *.043) (Fig. 2). Concerning clinical parameters, in our study population, the mean score (± SD) of GH was 46.9 ± 23, 47.6 ± 25.7 for pain-VAS and 50.5 ± 25.2 for patient-VAS. A strong statistical significant correlation was obtained among TAS 20 score and GH, pain-VAS and patient-VAS mean values (*P < *.0001 for each parameter) (Fig. 3A–C).

Finally, a trend of statistically significance was observed between concomitant steroid therapy and high TAS-20 score and between female sex and high TAS-20 score. No correlations among variables such as age, duration of disease, and comorbidities and alexithymia status were observed in our cohort.

## 4 Discussion

Inflammatory arthritis are chronic rheumatologic disorders with both social and psychiatric burden in addition to joint involvement. Moreover, the presence of comorbidities, involving also the psychiatric sphere, has been frequently associated to RA and PsA.^[21,22]^ The present study focused on an aspect of these disorders such as alexithymia that manifests itself as an inability to describe and identify emotions and may have an impact on disease activity and treatment efficacy.

Chronic pain, defined as pain lasting at least 3 months, is more complicated than acute pain, since it occurs in neurobiological, psychological, and social changes. The nosology of persistent pain is inconsistent and evolving. Some types of pain are tied to disease processes in specific tissues, including joint degeneration (osteoarthritis), inflammation (e.g., RA, inflammatory bowel disease), tumor growth (cancer pain), damaged nerves (neuropathic pain), or tissue anoxia (sickle cell disease).^[23]^ Few studies have been published in the field of alexithymia in inflammatory arthritis and its possible causes. A study conducted by Velasco and collaborators evaluated alexithymia, anxiety, depression, and emotional awareness, underling how all these parameters were more frequent and severe in patient with chronic rheumatological diseases than in healthy control. However, a limit of this study was that patient affected by fibromyalgia were included.^[24]^ Taylor and collaborators evaluated 2795 patients with RA in order to measure patients’ perceptions and pain management in RA. The majority of patients reported their RA as being somewhat-to-completely controlled, while only 12% in Europe and 15% in the United States reported not at all controlled or poorly controlled. As expected, patients with all levels of RA severity reported pain, the level of pain (mild, moderate, or severe) corresponding with reported RA severity. Patient satisfaction with arthritis pain was significantly correlated with satisfaction with other areas of health, as social activity, tension, mood and fatigue, underlining how relevant is a good manage of physical symptoms influences the psychological sphere.^[25]^

To the best of our knowledge, no studies have been conducted evaluating the presence of alexithymia in patient affected by PsA or by RA, in the absence of fibromyalgia.

In the present study, high TAS-20 scores were observed in patient with an active disease, typically characterized by the presence of inflammation and pain at joint level. As a matter of fact, clinimetric parameters such as GH index, pain-VAS, and patient-VAS have been statistically significantly associated to the presence of alexithymia. Furthermore, patient with high ESR and CRP values resulted positive to the presence of alexithymia, showing how a chronic condition of inflammation and pain, as in RA and in PsA, can conduce to an alteration of the psychiatric sphere.

Concerning gender, higher prevalence of alexithymia in female patients was expected, being women more susceptible to mood and personality changes. In this study, a trend of correlation among TAS-20 scores and female gender was found. Data concerning the presence of alexithymia in patients affected by RA and PsA are few in the literature, but the relation between inflammatory arthritis and central nervous system disorders has been well known; however, how they can influence each other is still unknown. A hypothesis takes in consideration the role of TNF (tumor necrosis factor) in the inflammatory process.^[1]^ This cytokine, strongly associated with the pathogenesis of RA and PsA, is able to interact with receptors expressed on the surface of the astrocytes, inducing them in a pro-inflammatory condition. TNF is also involved in some central nervous system pathologies such as Alzheimer.^[24]^ At the same time, TNF has a role in influencing the circadian clock, mediated by the *clock genes* (Cry1 and Tef), with a direct action on these genes.^[26]^ In particular, the abnormal expression of these genes, altered in cells stimulated by TNF, is associated with depression.

The main limits of our study were: the lack of a control group for the detection of alexithymia in healthy controls; however its prevalence in healthy people is well known in the literature; the relative small sample size of patients included in the single-group analysis when divided according to other variables as the presence of comorbidities or gender; further investigations on a larger cohort of patients affected by spondyloarthritis are awaited.

In conclusion, our study focused on the opportunity and the necessity to evaluate patient affected by rheumatological diseases, as RA and PsA, in a more comprehensive way, taking into consideration the presence of comorbidities and choosing a more appropriate treatment, which must include the control of all physical and psychological symptoms.

Further studies are needed, involving a more complete psychiatric assessment, with personality tests, in order to better understand how TAS-20 can be used in daily clinical practice for the detection of mood changes in patients affected by RA and PsA and the impact of alexithymia in patients’ outcomes and treatment efficacy.

### 4.1 Take home messages

- Inflammatory arthritis, as RA and PsA, are chronic rheumatologic disorders with both social and psychiatric burden.
- There is a strong relationship between inflammatory arthritis and central nervous system disorders.
- Alexithymia is a personality trait characterized by deficits in cognitive processing and regulation of emotions, related to the inflammatory status in RA and PsA patients.
- Alexithymia was also associated to pain and functional status indices in RA and PsA patients.
- Evaluate patients affected by RA and PsA in a more comprehensive way, including the psychiatric sphere may be useful for a more complete therapeutical management.

## Author contributions

**Conceptualization:** Maria Sole Chimenti, Elisabetta Greco, Roberto Perricone.

**Data curation:** Juna Hithj, Barbara Kroegler, Marina Talamonti.

**Formal analysis:** Paola Conigliaro.

**Methodology:** Paola Conigliaro, Paola Triggianese, Miriam Teoli, Marco Galluzzo, Barbara Kroegler.

**Project administration:** Giulia Lavinia Fonti, Elisabetta Greco.

**Resources:** Paola Triggianese, Miriam Teoli, Marco Galluzzo.

**Supervision:** Roberto Perricone.

**Writing – original draft:** Maria Sole Chimenti, Giulia Lavinia Fonti.

**Writing – review & editing:** Marina Talamonti, Roberto Perricone.
